# Life Skills Training Effectiveness on Non-Metastatic Breast Cancer Mental Health: A Clinical Trial

**DOI:** 10.5812/ircmj.8763

**Published:** 2014-01-05

**Authors:** Mina Shabani, Minoosh Moghimi, Reza Eghdam Zamiri, Fatemeh Nazari, Nouraddin Mousavinasab, Zahra Shajari

**Affiliations:** 1 Department of Psychiatry, Zanjan University of Medical Sciences, Zanjan, IR Iran; 2 Departments of Hematology and Oncology, Zanjan University of Medical Sciences, Zanjan, IR Iran; 3 Department of Radiation Oncology, Zanjan University of Medical Sciences, Zanjan, IR Iran; 4 Department of Statistics, Faculty of Medicine, Zanjan University of Medical Sciences, Zanjan, IR Iran; 5 Zanjan Metabolic Disease Research Center, Zanjan University of Medical Sciences, Zanjan, IR Iran

**Keywords:** Breast Neoplasms, Life, Skill, Education, Quality of Life

## Abstract

**Background:**

Patients with breast cancer are predisposed to some psychiatric symptoms and mental disorders due to their life styles or disease conditions. These problems cause patients to deal with daily stress, feeling guilty, anxiety, dysphoric mood, and impaired social relations. Such problems would lead to serious mental disorders.

**Objectives:**

Therefore, life skills training may help patients to cope better with their condition, and improve their mental health.

**Materials and Method:**

In an experimental study, 50 patients with breast cancer were selected randomly and assigned to 2 experimental and control groups. The experimental group attended life skills training classes for 10 weeks continuously (each class lasting 2 hours). Participants in both the experimental and control groups completed a GHQ-28 questionnaire form before the commencement of classes, and again after 2 weeks to 2 months of the course completion. T-test was used as the statistical method.

**Results:**

In life skills training group, depressive and anxiety symptoms, somatization disorders, sleep disorders and disorders of social functioning were significantly decreased (p<0.0001). These changes were not observed in the control group.

**Conclusions:**

The results showed that life skills training is an effective method in reducing symptoms of depression, anxiety, sleep and somatic disorders. Also, it would be useful in reducing problems of social dysfunction.

## 1. Background

Breast cancer is the most common type of cancer among women worldwide (1-3), which accounts for approximately one-fifth of all deaths in women aged 40–50 years (4). In Iran, its incidence was estimated to be 20 per 105 women, and one of every 10 women would develop breast cancer during her life (5-7). Treatment options include surgery, radiotherapy, and chemotherapy which lead to increased disease- free survival, better tumor response, and overall survival improving. On the other hand, cancer and therapies complications have confronted patients with terrifying psychological experiences and morbidities like anxiety, depression, and poor quality of life.

Previous reviews of the literature have indicated that psychological therapies and life skills training may help patients with cancer by increasing their knowledge about their disease and treatment, by improving their emotional adjustment, satisfaction, and their physical condition, and reducing treatment and disease-related symptoms (8-12). Besides improving conditional therapies for patients with breast cancer, tendency to use new psychological interventions is growing. One of these psychological packages is life skills training program defined by WHO as ability for adaptive and positive behaviors which enables individuals to deal effectively with the demands and challenges of everyday life, and consists of 10 abilities (13).

## 2. Objectives

We designed a clinical trial to examine the effects of life skills training on psychological distress and coping with primary breast carcinoma among Iranian women.

## 3. Materials and Methods

### 3.1. Subjects

Fifty subjects with diagnosed breast carcinoma in department and clinic of oncology in Vali-e-Asr hospital were selected. The study protocol was reviewed and approved by the Department of Internal Medicine and the Ethics Committee of Zanjan University of Medical Sciences, Iran. Eligibility criteria for the current study population were: 1) age younger than 65 years; 2) diagnosed breast carcinoma in stages I, II or III who completed standard therapy including mastectomy, chemotherapy, and radiotherapy and being under hormone therapy during the clinical trial. Exclusion criteria were mental disorders, dementia, psychosis or acute psychological disorder like major depression, or if they had cancer at another site. None of the subjects had received psychological consult before the study participation. Eligible subjects were informed for this psychosocial group intervention and life skill training study. They were informed that all cancer patients experience psychological distress, and this life skills training is useful for improving the quality of life of patients with breast carcinoma according to the same researched performed in other countries. All patients provided written informed consent before the assessment.

### 3.2. Intervention Protocol

Patients who wished to participate in the intervention and met the eligibility criteria were randomly categorized to either the experimental group or the control group (each containing 25 subjects) by using their birth certificate number. Demographic information of patients included age, education, number of children, occupation, income, and the duration of breast cancer were recorded in a questionnaire. Because previous studies have shown that individual intervention requires too much time and cost in comparison to group intervention (14-18), and group intervention to be as effective in solidarity and interaction between group and emotional draining (15, 19, 20), we chose a group model in this study.

We aligned 10 sessions, for 2 hours and lasting, totally, in 10 weeks. In these workshops 10 life skills and techniques (decision making, problem solving, creatively thinking, critically thinking, communication skill, interpersonal relationship, self-confidence, feeling empathy, emotion handling, and tension handling recommended by WHO) (18) and skill application in patients’ lives were taught by trained and qualified trainers during these workshops and observed by psychologists. At the end of each session, subjects were assessed about skill of that session, and trainers resolved their questions.

### 3.3. Measurement

The General Health Questionnaire-28 (GHQ) was designed by Goldberg DP (21), and its reliability and validity were assessed (15, 22), standardized for screening in Persian language, in Iran. It has four subscales: 1) somatization symptoms, 2) anxiety and sleeping disorders, 3) social functioning, and 4) depression (D). Each subscale contains 7 ‘here and now’ questions. Scoring system of GHQ questions was based on psychological discomfort (lowest score = 1) up to psychological health (highest score = 4). The total score of each question varies from 7 to 28, and the total range for score of General Health Questionnaire is estimated from 28 to 112. In this questionnaire, psychiatric symptoms and abnormal behaviors of patients were elicited. Subjects completed GHQ-28 just before the training workshop, at the end of 2 weeks education period, and 2 months after the completion of training courses. Lower scores indicated more impaired psychological condition.

### 3.4. Statistical Analysis

Statistical analysis was performed using the Statistical Package for Social Science (SPSS version 16). Mean values (± SEM), median, ranges are shown. Descriptive statistical methods were used where appropriate. Demographic and clinical characteristics and baseline psychological scores were tested by the Student T test. Preliminary analyses included descriptive and bivariate analyses (i.e. analyses of variance and to examine comparability between the groups on socio demographic, medical, and baseline QOL characteristics.

## 4. Results

This study was conducted among 50 patients with breast cancer in different stages of carcinoma I, II, III who had completed their standard therapy before psychological intervention; they were divided into 2 experimental and control groups randomly. The mean age was 46.7 ± 9.3 years in the intervened patients, compared to 45.7 ± 8.9 in the control subjects, with no significant difference (P = 0.714). Demographic and social characteristics of the 2 groups were summarized in Table 1. There was no difference between both groups regarding their occupation, education level, number of children, and monthly income. The mean time of illness awareness was 2.64 ± 1.22 years for the experimental group, and 2.68 ± 1.94 for the control group (P = 0.897).

GHQ-28 scores of 4 subtitles include somatization symptoms, anxiety and sleep disorders, social function disorder, and depression disorder (Table 2). 

**Table 1.** Demographic and Psychological Characteristics of Patients With Breast Cancer

Character	Experimental Group	Control Group	P Value
**Occupation**			0.384
House wife	23	21	
Employed	2	4	
**Education**			0.972
Illiteracy	2	2	
Elementary school	15	14	
High school	6	6	
University	2	3	
**Number of children**			0.57
2	4	4	
3	7	9	
4	8	7	
5	4	4	
6	2	1	
**Month income**			0.765
Under 200$	8	9	
Over 200$	17	16	
**Illness awareness, mean ± SD, y, n= 25**	2.64 ± 1.22	2.68 ± 0.94	0.897

**Table 2.** Scores for 4 GHQ-28 Subtitles of Experimental and Control Groups Before and After Life Skills Training

Subtitle	Experimental Group, n = 25			Control Group, n = 25		
	Before	2 Weeks After	2 Months After	Before	2 Weeks After	2 Months After
**Psychosomatic symptoms**	19	21.68	20.6	19.52	19	19.64
**Anxiety and sleep disorders**	13.28	19.52	19.36	19.28	19.65	22.28
**Social function disorder**	19.64	23.12	22.28	19.04	19.24	19.64
**Depression disorder**	13.76	16.28	16.20	12.64	12.76	12.2
**Total score**	65.68	80.6	78.4	70.48	70.46	73.76

**Table 3.** Comparison of Mean of Changes in 4 Subtitle Scores Prior, 2 Weeks, and 2 Months After Life Skills Training Program

Time and Groups, n = 25	Mean of Changes					
	Before and After 2 Weeks	P value	Before and After 2 Weeks	P value	2 Weeks and 2 Months After	P value
**Psychosomatic Symptom**	-	< 0.00001	-	< 0.00001	-	0.002
Experimental Group	2.68	-	1.6	-	1.08	-
Control group	-0.62	-	0.12	-	0.64	-
**Anxiety and sleep disorder**	-	< 0.00001	-	0.001	-	< 0.00001
Experimental Group	6.24	-	6.08	-	-0.16	-
Control group	0.36	-	3	-	2.64	-
**Social function disorder**	-	< 0.00001	-	0.001	-	0.02
Experimental Group	3.48	-	2.64	-	-0.84	-
Control group	0.2	-	0.6	-	0.4	-
**Depression disorder**	-	< 0.0001	-	0.0001	-	0.28
Experimental Group	2.52	-	2.44	-	-0.08	-
Control group	0.12	-	-0.044	-	-0.56	-
**Total score**	-	< 0.0001	-	< 0.0001	-	< 0.0001
Experimental Group	14.94	-	12.76	-	-2.16	-
Control group	0.16	-	3.28	-	3.12	-

Data analysis indicated that somatization symptoms score increased 2 weeks after the intervention significantly (P < 0.001), and this increment persisted after 2 months too (P < 0.00001). These differences were not observed in the control group. Anxiety and sleep disorders assessment of experimental group revealed a considerable increase in the score before skill training (13.2 ± 2) compared to 2 weeks after the training (19.5 ± 2) (P < 0.00001). Changes in anxiety and sleep disorders remained after 2 months (P < 0.001). After 2 weeks of life skills training workshop, social function disorder scale improved and reached 23.1 ± 1 (P < 0.0001), and after 2 months, this increase was statistically significant compared to the score before the intervention (P < 0.001).

**Figure 1.**
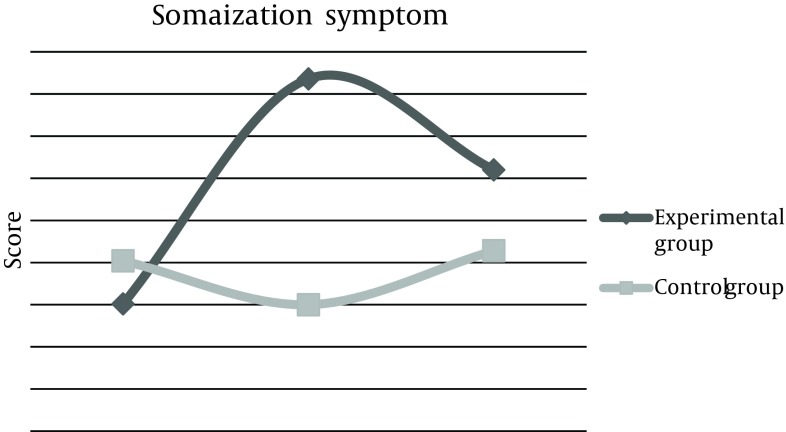
Comparison of Mean Score Changes in Psychological Symptoms (GHQ-28) Before and After Life Skills Training in Experimental and Control Groups

**Figure 2.**
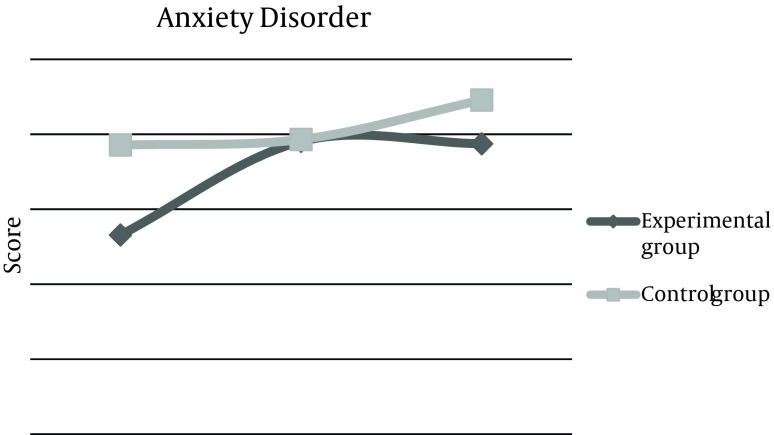
Comparison of Mean Score Changes in Anxiety Symptoms (GHQ-28) Before and After Life Skills Training in Experimental and Control Groups

**Figure 3.**
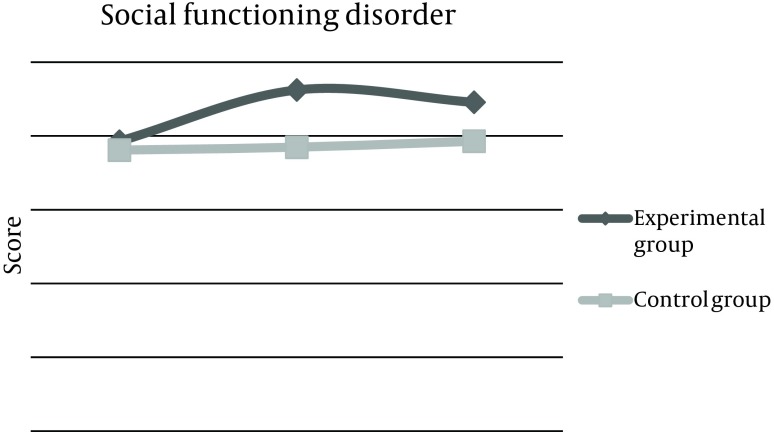
Comparison of Mean Score Changes in Social Functioning Disorder (GHQ-28) Before and After Life Skills Training in Experimental and Control Groups

**Figure 4.**
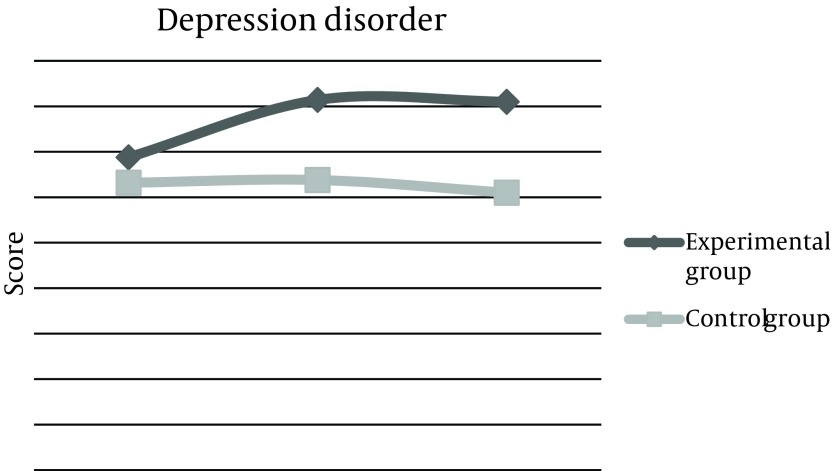
Comparison of Mean Score Changes in Depression Disorder (GHQ-28) Before and After Life Skills Training in Experimental and Control Groups

**Figure 5.**
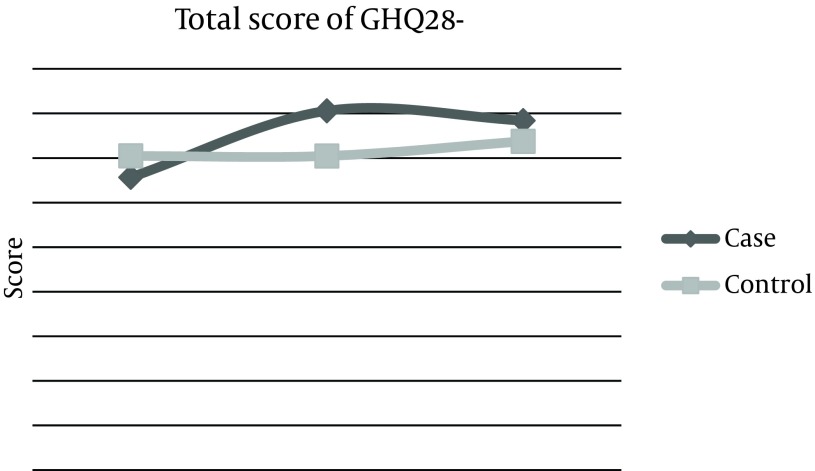
Comparison of Mean Score Changes in Total Score of GHQ-28 Questionnaire Before and After Life Skills Training in Experimental and Control Groups

Mean score of depression disorder phase before the experiment was 13.7 ± 4, 2 weeks after the experiment increased to 16.2 ± 4, and 2 months later, remained 16.2 ± 2. This score was 12.6 ± 1 for the control group at baseline, which did not change after 2 weeks and 2 months of reassessment. The total score of questionnaire in the experimental group was estimated 65.68 before the intervention, but increased up to 80.6 after 2 weeks, which was statistically significant and was persistent even after 2 months. These changes were not observed in the control group (Table 2). Also, means of changes were compared in three categories between the two groups. First; before and after 2 weeks of intervention, the second; before and 2 months after, and the third, comparison of changes between the results of after 2 weeks and after 2 months. Results demonstrated that differences between the means of changes were considerable in the experimental group (Table 3). These Mean scores of 4 subtitles before and 2 weeks and 2 months after life skills training workshop were illustrated in Figures 1, 2, 3, 4, and 5.

## 5. Discussion

Breast cancer is the most common type of cancer among women worldwide. For women, breast cancer is a terrifying disease due to a high mortality rate and body imaging distortion (1-3). Most patients with breast cancer have psychological reactions such as denial, anger, or fear toward their disease and treatment process. Many patients have psychiatric morbidities, especially anxiety and depressive disorders (23-25). Among psychiatric morbidities, anxiety and depressive disorders are the two disorders commonly found in patients with breast cancer. The range of anxiety disorder prevalence in breast cancer varied from 1 to 49 % (26), while depressive disorder ranged from 1.5 to 46 % (26, 27).

 In the recent years, there has been increasing interest in various aspects of mental health. Also, it is considered that psychosocial intervention could reduce the morbidity of patients breast carcinoma, improve the quality of life of patients with cancer, and its effects have been evaluated over the past 2 decades (21, 28-34). Health promotion is defined as ‘any deliberate intervention which seeks to promote health and prevents disease disability’ (35-38). WHO then defined a developed training program, with the aim of mental health promotion, named life skills. It means ability for adaptive and positive behavior which enables individuals to deal effectively with the demands and challenges of everyday life (13). The main purpose of life skills training is to promote healthy lifestyles through skills training.

These following life skills (recommended by WHO) (18) are:

1. The ability to make decisions helps people assess their options and carefully consider the different consequences that can result from their choices.
2. The ability to solve problems helps people find constructive solutions to their problems. This skill can significantly reduce anxiety.
3. The capacity to think creatively helps people make decision and solve problem, and look beyond their personal experience.
4. The capacity to think critically helps people analyze information along with their own experiences.
5. The ability to communicate effectively helps people express their feelings, needs, and ideas to others.
6. The ability to establish and maintain interpersonal relations helps people interact positively with people whom they encounter daily, especially family members.
7. Knowledge of self is the capacity of people to know who they are, what they want and do not want, and what does and does not please them which helps people recognize stressful situations.
8. The capacity to feel empathy is the ability to imagine what life is like for another person in a very different situation. It helps people to understand and accept diversity, and it also improves interpersonal relations between diverse individuals.
9. The ability to handle emotions enables subjects to recognize their emotions and how they influence their behaviors.
10. The ability to handle tension and stress (34, 39).

Efficacy of life skill training and psychological intervention depends on many variables such as (40) patients’ clinical and demographic characteristics like cancer stage and course of the disease, medical treatment, age, and gender, and educational level, income, occupation (41), and duration of psychosocial interventions; for example results of a meta-analysis indicated that the most important moderating variable was duration of psychosocial intervention and durations over 12 weeks would be more effective significantly rather than shorter durations (42). Moreover, the methodological quality of intervention studies; choosing a control groups, randomization status of treatment conditions, or documentation of experimental and statistical designs and procedures can lead to a higher mental health level.

 Studies on the effectiveness of life skills training either on the normal population quality of life or subjects with other bodily problems confirmed efficacy of these educations. For example working women predisposed to many psychiatric symptoms or disorders were attended 1-2 sessions of life skills training weekly for 10 weeks. The results of this study showed that life skills training can be an effective method in reducing anxiety, sleep and somatic symptoms of subjects (43). Or results of quality of life evaluation in 40 patients with coronary heart aged 35-65 years, having bypass for the first time after life skills training program showed that group life skills training is effective to decrease anxiety and depression in patients with coronary diseases after coronary bypass surgery (44). In the recent study, we evaluated efficacy and psychological power of life skills training program on improvement of non-metastatic breast cancer quality of life. As described in materials and methods, GHQ-28 questionnaire was designed in a way as lower scores indicate poor mental and physical condition, and a higher score expresses a better, healthy mental status. In this study, in spite of training the ways of increasing self-esteem and controlling feelings in training sessions, but we did not measure their effects on quality of life. As indicated in this study, there were not significant differences in 4 GHQ-28 items including 1) somatization symptoms, 2) anxiety and sleeping disorders, 3) social functioning disorder, and 4) depression between the experimental and control groups before life skills training intervention. After 2 weeks of psychological intervention, we found a remarkable reduction in somatization symptoms, anxiety and sleeping disorders, social functioning disorder and depression symptoms in the experimental group compared to the control and before the intervened condition. Also, life skills training effectiveness on quality of life and anxiety and depression reduction remained after 2 months of reassessment.

The result of a meta-analysis summarized the results of 37 published, controlled studies that investigated the effectiveness of psychosocial interventions on quality of life (QoL) in adult patients with cancer, and findings supported the usefulness of psychosocial interventions for improving quality of life in adult patients with cancer (45). Three hundred female patients with breast cancer, aged above 18 years old from the Surgical Outpatient Department, King Chulalongkorn Memorial Hospital were evaluated in a study from December 2006 to May 2007, and showed that anxiety and depressive disorders are the two common psychiatric disorders in patients with breast cancer. Improving patients’ social support and raising patient’s coping skills reduced the patients’ psychological stress and psychiatric morbidities (39). 

Japanese scientists conducted a 6-week, psychosocial group intervention on patients with breast cancer with the followings inclusion criteria; age younger than 65 years, lymph node metastasis positive and/or histologic or nuclear grade 2–3, and undergone surgery within the past 4–18 months from the beginning of study. The intervention consisted of health education, coping skills training, stress management, and psychological supports. Patients were evaluated for psychological distress by the Profile of Mood States (POMS), Mental Adjustment to Cancer (MAC) scale, and Hospital Anxiety and Depression (HADS) scale. They inferred that short term psychosocial intervention produces significant long term enhancing of quality of life in Japanese patients with primary breast cancer (46). Thirty-six patients with non-metastatic breast cancer were assessed in G. Marchioro et al. investigation. Patients received either psychological intervention (weekly cognitive individual psychotherapy and bimonthly family counseling) or standard follow-up. Personality (16-PF and IIQ), quality of life (FLIC), and depression (BDI) scores were the endpoints for this study, and evaluated in the patients at diagnosis, and up to 9 months after the diagnosis. This study indicated that cognitive psychotherapy and family counseling improved both depression and quality of life indices compared to the control group (30).

Therefore, by citing to the previous researches findings and this study; psychological consultation therapies are recommended for patients with cancer because they expect these therapies to cure their cancer or to improve their recovery and both patients and oncologists would be moderately to very satisfied with the results of psychological therapies. In conclusion, many investigations concluded that psychological therapies might help patients with cancer in various ways, ranging from reducing the side effects of cancer treatments to improving patients’ immune function and longevity.
